# The *Bacillus anthracis *cholesterol-dependent cytolysin, Anthrolysin O, kills human neutrophils, monocytes and macrophages

**DOI:** 10.1186/1471-2180-6-56

**Published:** 2006-06-21

**Authors:** Elise M Mosser, Richard F Rest

**Affiliations:** 1 Department of Microbiology and Immunology, Drexel University College of Medicine, 2900 Queen Lane, Philadelphia, USA

## Abstract

**Background:**

*Bacillus anthracis *is an animal and human pathogen whose virulence is characterized by lethal and edema toxin, as well as a poly-glutamic acid capsule. In addition to these well characterized toxins, *B. anthracis *secretes several proteases and phospholipases, and a newly described toxin of the cholesterol-dependent cytolysin (CDC) family, Anthrolysin O (ALO).

**Results:**

In the present studies we show that recombinant ALO (rALO) or native ALO, secreted by viable *B. anthracis*, is lethal to human primary polymorphonuclear leukocytes (PMNs), monocytes, monocyte-derived macrophages (MDMs), lymphocytes, THP-1 monocytic human cell line and ME-180, Detroit 562, and A549 epithelial cells by trypan blue exclusion or lactate dehydrogenase (LDH) release viability assays. ALO cytotoxicity is dose and time dependent and susceptibility to ALO-mediated lysis differs between cell types. In addition, the viability of monocytes and hMDMs was assayed in the presence of vegetative Sterne strains 7702 (ALO+), UT231 (ALO-), and a complemented strain expressing ALO, UT231 (pUTE544), and was dependent upon the expression of ALO. Cytotoxicity of rALO is seen as low as 0.070 nM in the absence of serum. All direct cytotoxic activity is inhibited by the addition of cholesterol or serum concentration as low as 10%.

**Conclusion:**

The lethality of rALO and native ALO on human monocytes, neutrophils, macrophages and lymphocytes supports the idea that ALO may represent a previously unidentified virulence factor of *B. anthracis*. The study of other factors produced by *B. anthracis*, along with the major anthrax toxins, will lead to a better understanding of this bacterium's pathogenesis, as well as provide information for the development of antitoxin vaccines for treating and preventing anthrax.

## Background

*Bacillus anthracis*, a spore-forming, aerobic, Gram-positive bacterium is the causative agent of the disease anthrax. The fact that the *B. anthracis *spores are highly lethal, inexpensively made, easily produced and disseminated as weapons of terror, necessitates the need for a better understanding of the pathogenic mechanisms and virulence factors of this organism using human cells [1-3]. In all forms of anthrax, gastrointestinal, pulmonary, or cutaneous, *B. anthracis *spores are phagocytosed by residing intestinal, alveolar, or skin macrophages where they germinate into vegetative bacilli. The macrophages then travel to the lymph nodes where the vegetative *B. anthracis *must escape the macrophage's vacuole into the cytoplasm and eventually the macrophage itself. Bacilli multiply in the lymphatics and enter the bloodstream, where they can reach numbers of 10^7 ^to 10^8 ^per milliliter [1].

*Bacillus anthracis *vegetative bacteria express virulence factors that are encoded by two virulence plasmids, pXO1 and pXO2 [1,4,5]. pXO1, the toxin gene-bearing, regulatory plasmid, encodes the known components of *B. anthracis *exotoxins: lethal factor (LF), edema factor (EF) and protective antigen (PA). Together with pXO2, the capsule producing plasmid, anthrax toxins are thought to be responsible for the high morbidity and pathology of anthrax during the late stage of disease and have been a primary focus of *B. anthracis *research for decades. Although anthrax lethal toxin (LT) may contribute significantly to septicemia and death of the host, other virulence factors may be important in establishing infection and may contribute to disease [2,6,7].

*In vitro *and *in vivo*, vegetative *B. anthracis *express and secrete a protein which is member of the cholesterol-dependent cytolysin (CDCs) family of cytolysins. The protein, which we have named Anthrolysin O (ALO), acts as a hemolysin against human red blood cells and is differentially regulated by environmental signals [8-10]. Members of the CDC family of cytolysins are secreted and bind cholesterol and other molecules in the host cell plasma membrane forming large pores which range in diameter from 20 to 30 nm [11-13]. The formation of pores in cholesterol-containing membranes confers this family of toxin's hemolytic and cytolytic properties. CDCs are intimately involved in the pathogenesis of more than 20 Gram-positive species including *Arcanobacterium pyogenes, Clostridium perfringens, Bacillus cereus, Listeria monocytogenes, Streptococcus pyogenes and Streptococcus pneumoniae *[14-18]. At high concentrations, CDCs function as cytotoxins, lysing host immune cells; Perfringolysin O, PFO, is cytolytic to macrophages; Pneumolysin, PLY, lyses murine macrophages and bovine PMNs, and; SLO of *S. pyrogenes *lyses human PMNs [19-22]. Many of these pathogens secrete CDCs in sub-lytic amounts that function to modulate phagocytic cell function by inhibiting chemotaxis, the oxidative burst, or phagocytosis, and activate complement [12,15,23-27]. Most recently CDCs, including ALO, have been described as TLR4 agonists [28-30].

During *B. anthracis *infection the events associated with phagocyte membrane disruption, phagocyte death, and escape of vegetative bacilli from the phagocyte have yet to be fully elucidated. This prompted us to examine the role that ALO might play as a cytolysin against human phagocytes. We hypothesize that ALO can be defined as a new *B. anthracis *virulence factor, which has the ability to lyse human monocytes, human monocyte-derived macrophages (hMDMs), lymphocytes and neutrophils (PMNs).

Our studies reveal by trypan blue exclusion assay and by the release of LDH, that purified recombinant ALO (rALO) is cytolytic to THP-1 monocytes, freshly isolated PMNs, lymphocytes, hMDMs, ME-180, Detroit 562, and A549 epithelial cells. Furthermore, using Sterne strain 7702, an ALO-negative mutant, and a complemented strain expressing the native *alo *gene, we show that viable, vegetative *B. anthracis*, or their supernatants, lyse THP-1 s and macrophages in a dose dependent manner. We demonstrate that the death of the leukocytes is dependent upon ALO.

## Results

### Recombinant ALO lyses human primary phagocytes and lymphocytes in a dose and time dependent manner

**Figure 1:**
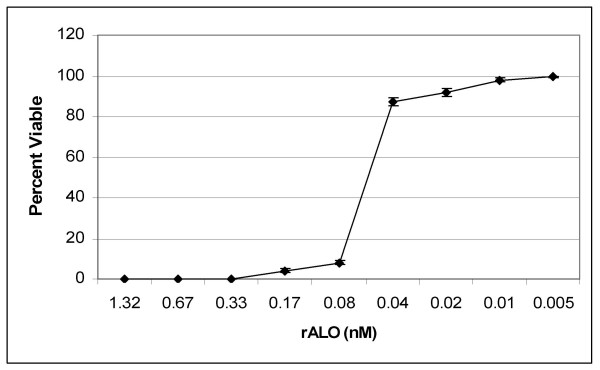
**Recombinant ALO lyses primary human neutrophils in a dose dependent manner**. Percent viability of PMNs measured by trypan blue after 10 min incubation with various concentrations of rALO in serum free conditions. The means and standard deviations of two independent experiments, each performed in duplicate are shown (Spearman correlation is -0.842, p = 0.004).

Overnight growth supernatants from Sterne strain 7702 and rALO are hemolytic to human [8] and mouse red blood cells (data unpublished). However, we did not know if ALO was cytolytic to human leukocytes. Because of the lethal effects of other CDCs on human and mouse phagocytes, we hypothesized that ALO would act similarly, lysing human phagocytes and lymphocytes [19-22]. We assayed for the ability of purified rALO to lyse freshly isolated human neutrophils by incubating neutrophils under serum-free conditions with a range of rALO concentrations. Addition of trypan blue to the cells and subsequent blind counting of at least 100 cells, show that as little as 4 ng/ml of rALO, which is approximately 0.070 nM, lysed neutrophils (Fig. 1). Lysis of the cells occurred within 30 min and little difference was seen at later time points. Addition of heat-inactivated 10% FBS before the addition of rALO inhibited lysis at all concentrations tested. Likewise, when 1 μg/ml of free cholesterol was pre-incubated with rALO before addition to the PMNs, viability of the PMNs remained the same as controls (data not shown).

**Figure 2:**
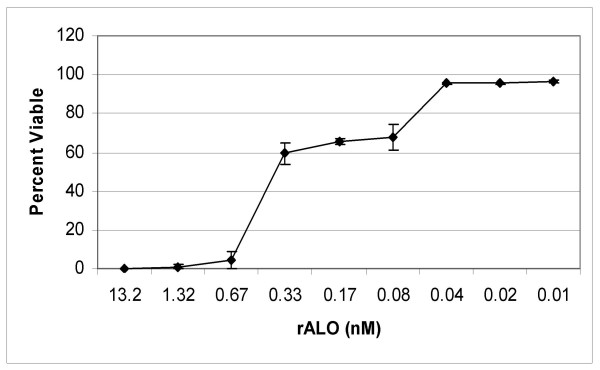
**Recombinant ALO lyses human monocytic THP-1 cells in a dose-dependent manner**. Percent viability of THP-1 monocytes measured by trypan blue after 30 min incubation with various concentrations of rALO in serum free conditions. Results show that 50% of THP-1s are lysed by 0.35 nM of rALO. The means and standard deviations of at least three independent experiments, each performed in duplicate, are shown (Spearman correlation is -0.870, p = 0.002).

To determine if human monocytes and macrophages were as susceptible to rALO mediated lysis as human neutrophils, we assayed the death of the human monocyte line THP-1 and hMDMs after incubation with rALO. Viability was measured by trypan blue exclusion and verified using an LDH based cytotoxicity assay. In the absence of serum 17.5 ng/ml, or 0.30 nM, of rALO caused 50 % lysis of THP-1s. These results indicate that PMNs are 5 times more sensitive to ALO mediated lysis than are THP-1s (Fig. 2).

**Figure 3:**
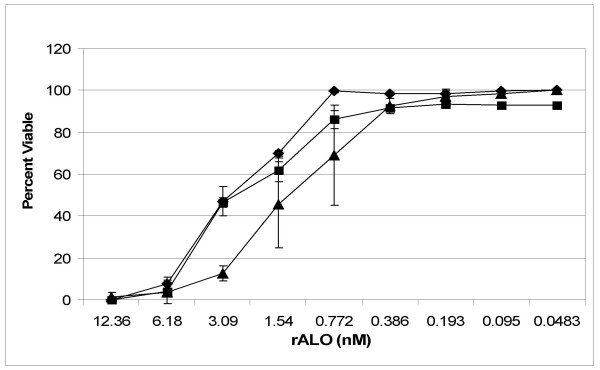
**Recombinant ALO lyses human monocyte derived macrophages (hMDMs) in a dose dependent manner**. Percent viability of primary human monocyte derived macrophages after 30 min incubation at 37°C with varying concentrations of rALO in serum free conditions. The means and standard deviations of three independent experiments performed in duplicate are shown. Each experiment was performed with hMDMs from different donors (represented by ▲, ◆, ■) (▲, Spearman correlation is -0.917, p = 0.001. ◆, Spearman correlation is -0.962, p < 0.001. ■, Spearman correlation is -0.852, p = 0.015).

Macrophages are thought to be the primary cells in/on which *B. anthracis *spores germinate, thus we sought to determine the cytolytic effect of rALO on primary hMDMs that had been differentiated for 5 days with M-CSF. After incubation for 30 min with rALO, in the absence of serum, the viability of hMDMs was assayed by trypan blue dye exclusion. In dose-response experiments, rALO was cytolytic to hMDMs (Fig. 3). As seen with human neutrophils and THP-1 monocytes, hMDMs are differentially lysed by rALO, requiring approximately 100 ng/ml, or 1.8 nM rALO to cause 50 % lysis.

**Figure 4:**
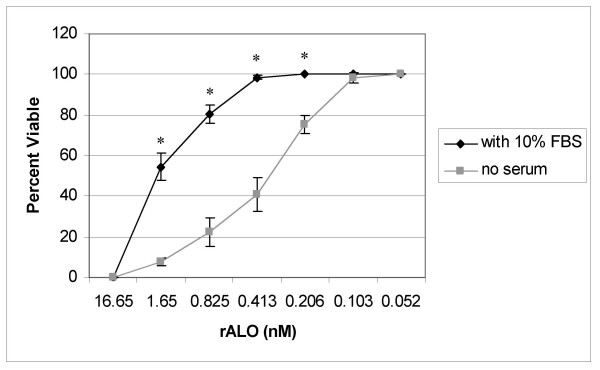
**Recombinant ALO lyses primary human lymphocytes in a dose dependent manner**. Percent viability of lymphocytes measured with trypan blue after 30 min incubation with varying concentrations of rALO at 37°C with no serum or heat-inactivated 10%. The means and standard deviations of at least three independent experiments, each done in duplicate, are shown (Spearman correlation is -0.964, p < 0.001). Addition of 10% serum significantly inhibits ALO-mediated lymphocyte lysis (*p = 0.001 as calculated by student's unpaired t-test).

Lymphocytes, although not cells of focus in *B. anthracis *infection, were also tested for their susceptibility to rALO lysis after observing different degrees of cell cytotoxicity depending upon the cell type assayed. Much like THP-1s, total lymphocytes isolated from human blood were lysed by rALO in a dose dependent manner in both the absence and, significantly to a lesser extent, in the presence of heat-inactivated 10% FBS (p = 0.001, student's un-paired t-test) (Fig. 4). Approximately 25 ng/ml, or 0.44 nM, of rALO was needed to cause 50 % lysis of human lymphocytes.

After establishing rALO's ability to lyse phagocytes and lymphocytes and observing their varying susceptibility to rALO-mediated lysis, we were prompted to investigate rALO's ability to lyse epithelial cells. Cells of the epithelial cell line ME-180 and Detroit 562 were incubated with varying concentrations of recombinant ALO for 30 min. The plate was briefly centrifuged and the supernatants used to determine percent viability using LDH release. Surprisingly, our results show that a much greater rALO concentration, almost 1 μg/ml, is required to cause 50 % lysis of ME-180 cells and Detroit 562 cells in the absence of serum (data not shown). There was no decrease in ME-180 epithelial cell viability even after a 3 h incubation. Similar results were observed using trypan blue exclusion. In contrast, when A549 lung epithelial cells were assayed for viability, approximately 50 ng/ml, or 0.88 nM of rALO was needed to cause 50% lysis (data not shown).

### Native ALO lyses human monocytes and macrophages

**Figure 5:**
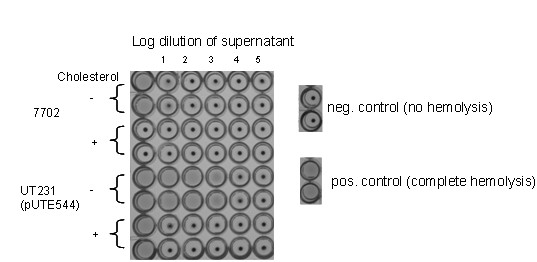
**Hemolytic activity of BHI growth supernatants of *B. anthracis *strains 7702, the parent ALO+ strain, and UT231(pUTE544), the ALO knockout strain complemented in trans with a plasmid bearing the *alo *gene**. UT231 (pUTE544) produces about 1,000-fold more ALO. Hemolytic activity is reduced upon the addition of 1 μg/ml cholesterol. Results are representative of at least three independent experiments done in duplicate.

**Figure 6:**
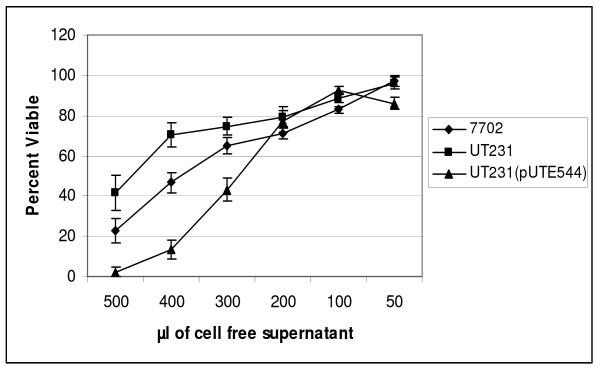
**Cell free supernatants from *B. anthracis *secreting ALO lyse human monocyte derived macrophages significantly more than the ALO knockout strain, UT231, in a dose dependent manner**. Percent viability of hMDMs from three different donors measured by trypan blue after the addition of filter sterilized *B. anthracis *supernatants in presence of heat-inactivated 10% FBS. The 3 h time point is shown, as little lysis was observed at earlier time points. Results are representative of results from three different experiments. (Dose, p < 0.001, strain, p < 0.001, dose by strain interaction, p = 0.044, as calculated by 2-way ANOVA. Tukey test showing significant differences between UT231/7702, UT231/UT231(pUTE544), 7702/UT231(pUTE544), p < 0.001)

To determine the role of ALO in *B. anthracis*-mediated cytotoxicity we investigated the ability of *B. anthracis *growth supernatants to lyse hMDMs. We asked the question whether native, secreted ALO lysed phagocytes in a similar manner to rALO. We have quantified the amount of ALO present in the supernatant of the ALO-overproducing strain, UT231 (pUTE544), by ELISA and hemolysis assays using a standard curve of rALO (n = 3, data not shown). Supernatant of UT231 (pUTE544) grown in BHI contains 1.38 ± 0.12 μg/ml ALO. Hemolysis assays revealed that the parent strain, 7702, grown under the same conditions, produces approximately 1,000-fold less ALO. Hemolysis is abrogated by the addition of 1 μg/ml of free cholesterol, indicating that the hemolysis is caused by ALO [8] (Fig. 5). Using this knowledge, late exponential phase cell free supernatants from the wild-type strain, 7702, were compared to supernatants obtained from the ALO knockout strain, UT231, and the complemented overproducing strain, UT231 (pUTE544), in their ability to lyse hMDMs. We hypothesized that strains producing ALO would be more cytolytic to hMDMs than the ALO knockout strain. Supernatants of strains 7702 and UT231 (pUTE544) caused significantly greater amounts of cytolysis of hMDMs than the ALO knockout strain UT231 (Fig. 6). Some cytolytic variability is seen from blood donor to blood donor, however overall lytic death occurs in a dose and strain, i.e. ALO dependent manner (p < 0.001, 2-way ANOVA, p < 0.001, Tukey test). Thus, native ALO, in addition to rALO, lyses human macrophages.

### *Bacillus anthracis *secreting ALO lyses human monocytes and macrophages

**Figure 7:**
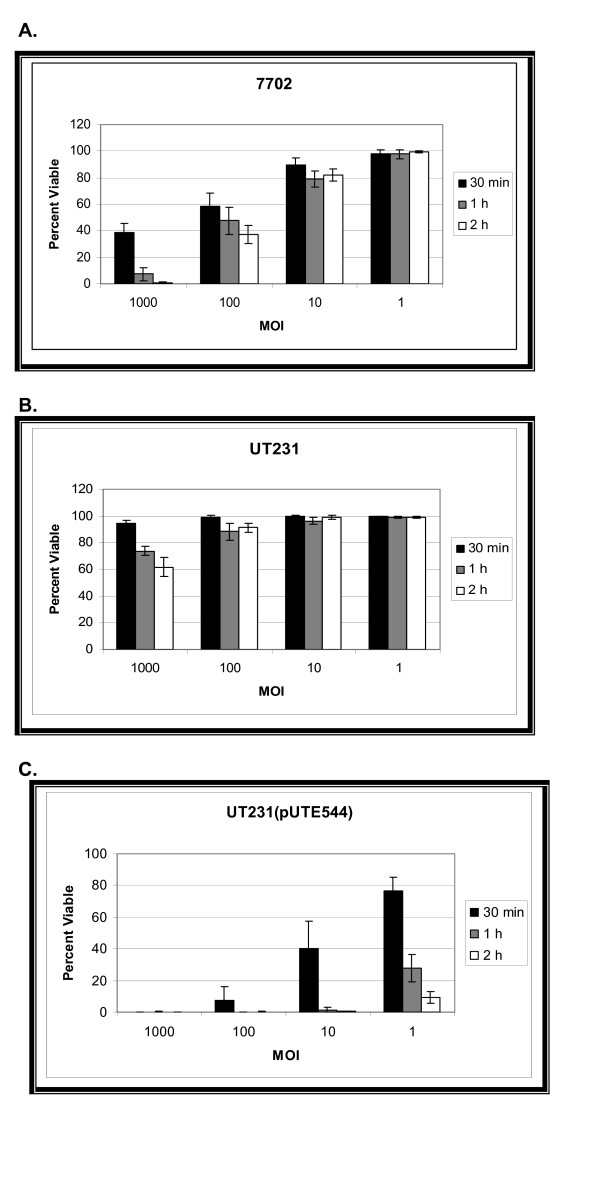
***Bacillus anthracis *strains secreting ALO lyse THP-1 monocytes in a dose and time dependent manner**. Percent viable THP-1 s in the presence of *B. anthracis *Sterne strain (A.)7702 (ALO+), (B.) UT231 (ALO-) and (C.) the complemented over-producer, UT231 (pUTE544) (ALO++) at MOIs of 1000, 100, 10 and 1 in serum free conditions. At time points, aliquots were mixed with 0.25 % trypan blue. Results are average ± standard deviations of three experiments, each performed in duplicate. (MOI of 100, 30 min, 1 h, 2 h, p < 0.001,1 way ANOVA. p < 0.001 for UT231/7702, UT231/UT231 (pUTE544), 7702/UT231 (pUTE544), Tukey test. MOI of 10, 30 min, 1 h, 2 h, p < 0.001,1 way ANOVA. 30 min, p < 0.001 for UT231/UT231 (pUTE544) and 7702/UT231 (pUTE544), Tukey test. 1 h and 2 h, p < 0.001 for UT231/7702, UT231/UT231 (pUTE544), 7702/UT231 (pUTE544), Tukey test.

Similarly, to determine if ALO played a role in the ability of live *B. anthracis *to lyse phagocytes, *B. anthracis *strains 7702, UT231 (ALO-), and UT231 (pUTE544) (ALO++) were grown to late log phase in BHI, washed and incubated with cells of the human monocytic cell line THP-1. Our results show that only bacteria expressing ALO completely lyse THP-1s in the absence of serum. Statistical analyses at a MOI of 100 at the 30 min, 1 h and 2 h timepoints show that 7702 and UT231 (pUTE544) lyse significantly more THP-1s than the ALO knock-out, UT231 (p < 0.001, 1-way ANOVA and p < 0.001, Tukey test (Fig. 7). Similar significance was also observed at a MOI of 10 after 1 h and 2 h. The decrease in viability by UT231 (ALO-) at an MOI of 1000, and to a lesser extent at MOI of 100, was noted, perhaps due to the effects of the major anthrax toxins or unknown toxins. However, an ALO-dependent dose and time response was observed when THP-1 cells were incubated with the ALO over-producing strain and 7702 over the 2 h time course examined. Therefore, addition of whole bacteria secreting ALO reproduced the observed cytolytic effects seen with purified recombinant protein.

### Addition of cholesterol abrogates ALO cytotoxicity of human monocytes

The presence of free cholesterol inhibits ALO-mediated lysis [8]. To support our hypothesis that ALO causes the observed cytolysis of THP-1 monocytes, cholesterol inhibition studies were performed. At an MOI of 1000 bacteria:monocyte the lysis caused by *B. anthracis *7702 (ALO+) and the complemented strain expressing ALO, UT231 (pUTE544) (ALO++) at 5 min was completely inhibited by pre-incubation of monocytes with heat-inactivated 10% FBS (Table 2). Similar results were seen using 1 μg/ml cholesterol (data not shown). After 1 h incubation insufficient amounts of cholesterol were present to inhibit the lysis caused by UT231 (pUTE544). These results are consistent with the inhibition of ALO's hemolytic activity by 1 μg/ml cholesterol in hemolysis assays and show that the observed *B. anthracis *cytotoxicity is a direct result of native ALO (Fig. 5).

## Discussion

*In vitro*, under nutrient rich growth conditions, and *in vivo B. anthracis *expresses and secretes a protein that is a member of the CDC family of cytolysins, which we have named ALO [8,9]. CDCs are closely involved in the pathogenesis of several Gram-positive bacteria, acting both lytically to lyse cells as well as sub-lytically, modulating the immune response and basic immune cell function [14-19,23-26,29,31]. CDCs, including ALO, stimulate through TLR4. Interestingly, ALO in conjunction with LT, at sub-lytic concentrations, is responsible for inducing apoptosis of macrophages. Furthermore, as a TLR4 agonist, ALO has the ability to induce an inflammatory response [29]. During the course of *B. anthracis *infection, the local concentration or expression of ALO likely varies, thus we hypothesized that ALO may act similarly to other CDCs and lyse human phagocytes, lymphocytes and epithelial cells. The secretion of a CDC by *B. anthracis *under certain growth conditions and the ability of this CDC to lyse human leukocytes may have implications regarding our understanding of basic *B. anthracis *pathogenesis.

Our results demonstrate that ALO causes cytolysis of primary human macrophages, neutrophils, lymphocytes and cells of the THP-1 human monocyte cell line in a dose and time dependent manner. Purified rALO, native ALO and *B. anthracis *expressing ALO all lyse human leukocytes. The observed cytotoxicity correlates with the proposed mode of action of ALO, namely, a multiunit accumulation of ALO molecules within the membrane of cells resulting in pore formation. Leukocyte and lymphocyte lysis by ALO occurred within 30 min, which is indicative of necrotic death. Likewise, when rALO-treated cells were assayed for apoptotic death by ethidium bromide/acridine orange staining, necrosis, but no apoptosis was evident (data not shown).

ALO cytotoxicity varied significantly in the different human cell types tested. PMNs were the most sensitive to ALO mediated lysis with 50 % lysis occurring at just 0.070 nM ALO. THP-1 monocytes, lymphocytes and monocyte-derived macrophages were 5.0, 6.25 and 25.0 times more resistant to ALO than were PMNs, respectively. Remarkably, in similar conditions, ME-180 and Detroit 562 epithelial cells were 250 times more resistant to ALO-mediated lysis than PMNs (Table 1). These differences in sensitivities between cell types are presently under study.

Like rALO, ALO-producing *B. anthracis *strains were cytolytic for human phagocytes. Cytotoxicity was abrogated by addition of cholesterol, confirming that lysis by *B. anthracis *is ALO-dependent under the conditions tested. Cholesterol is required for the binding of CDCs to cell membranes, and addition of free cholesterol acts as a competitive inhibitor for CDC binding and pore formation [32]. It is important to note, however, that intermedilysin does not use cholesterol as its receptor. Instead, glycosyl-phosphatidylinositol-linked membrane protein human CD59 is required for pore formation [11]. In cytotoxicity assays in which Sterne strain 7702, the over-expressing UT231 (pUTE544), or the ALO- strain UT231 were incubated with THP-1 monocytes, only bacteria producing ALO caused a dose and time dependent lysis of the cells (Fig. 7).

We have previously shown that during late exponential phase maximal amounts of ALO are secreted by *B. anthracis*, thus we assessed hMDM lysis by native ALO using bacterial growth supernatants[8]. When hMDMs were incubated with cell free late exponential phase growth supernatants from the parent strain 7702, a defined ALO knockout UT231, or the over-expressing ALO strain UT231 (pUTE544), significantly less cytotoxicity was observed with UT231 supernatants compared to supernatants of the parent and overproducing strains, indicating that ALO is the major factor contributing to the cytotoxicity of these phagocytes (p < 0.001, 2-way ANOVA and p < 0.001, Tukey test) (Fig. 6). These results strongly indicate that ALO is responsible alone or in conjunction with other toxins for the observed lysis. The death observed when UT231 (ALO-), supernatants were incubated with the hMDMs may be attributed to the major toxins, LT and ET. Overall, these data support our hypothesis that ALO at lethal concentrations may be involved with anthrax pathogenesis by lysing human neutrophils and monocytes/macrophages or by rendering them non-functional.

The mechanism responsible for the differential susceptibility of the human phagocytes and epithelial cells to ALO is largely unaccounted for and remains an interest of our lab. In cholesterol depletion experiments, lysis of THP-1s and mouse macrophages RAW 264.7 cells is abrogated after the addition of methyl-β-cyclodextrin, suggesting that in these cell types cholesterol is an important receptor for the pore forming toxin (in press) [33]. It may be hypothesized that a difference in membrane cholesterol composition exists among the cell types tested. Streptolysin O and pneumolysin and have shown different sensitivities to macrophages, monocytes and epithelial cells, respectively. These differences have been hypothesized to be the result of basic membrane composition and lipid raft content, however there are many factors that could contribute to differences in cell sensitivity [22,34]. ME-180 and Detroit 562 epithelial cells may be resistant to lysis even in the absence of free cholesterol due to carbohydrate moieties in the cell membrane that could impede pore formation. On the other hand, perhaps there is an unidentified protein receptor necessary for pore formation in these cells. We also observed differences between Detroit 562 and A549 epithelial cells, which reside in the pharynx and lung, respectively. These epithelial cells represent those that *B. anthracis *would likely come in contact with during inhalational anthrax. The observed differences in cell sensitivity are currently under study. Importantly, ALO has recently been identified as one of the hemolytic virulence factors secreted by *B. anthracis that *is responsible for increasing shedding of Synd1 and E-cadherin from epithelial cells, compromising the epithelial barrier integrity and perhaps leading to dissemination of infection [10].

Differential cell susceptibility may have implications regarding ALO's importance in *B. anthracis *pathogenesis. Perhaps PMNs, which are cells known to be first responders during bacterial infections, are quickly eliminated by ALO secreted by *B. anthracis*. Conversely, ALO may be less cytolytic to macrophages, a primary cell of *B. anthracis *pathogenesis, allowing spores to germinate and *B. anthracis *to persist for a greater length of time inside macrophages. Although we do not know the amount of ALO expressed by *B. anthracis *within macrophages or when growing in human blood, Klichko, et al. have noted that ALO is expressed at the early stages of infection within mouse macrophages by vegetative bacilli after spore germination [9]. Additionally, ALO mRNA has been detected in the spleens of *B. anthracis *infected mice, indicating that the gene is expressed *in vivo *(personal communication, T. Koehler). Furthermore, antibodies against ALO have been found in mice infected with Sterne strain *B. anthracis *spores, indicating that ALO is produced *in vivo *in concentrations high enough to elicit an immune response [10].

The cytotoxic role of ALO in anthrax pathogenesis, if any, has thus far not been elucidated. However, this study has shown that ALO, like its CDC counterparts, is able to lyse phagocytes. In murine models of pulmonary anthrax, disseminated anthrax results in pathological lesions in the spleen, ranging from mild to severe necrotizing splenitis. Lung associated lymph nodes also undergo complete cytolysis [35]. Whether this cytolysis is caused by LT, or other toxins, remains to be delineated. ALO most likely acts locally, rather than systemically, affecting PMN recruitment, macrophage function, or it may confer bacteria an advantage while in or near phagocytes. We have shown that fewer than 100 molecules of rALO are required to cause 100% lysis of human erythrocytes [8]. This suggests that *B. anthracis *would not have to secrete much ALO within the phagocytic vacuole, within the macrophage cytoplasm, or locally in order to initiate a biological response or death of the phagocyte [36]. ALO may be important in the *B. anthracis *macrophage interface whether it be from without or from within macrophages either before or after phagocytosis of the bacilli occur. Dixon, et al. suggested that escape from the macrophage is pXO1 independent and that a gene in the chromosomal DMA may serve to allow the escape from the macrophage [6]. Furthermore, lysis of host cells by CDCs helps bacteria avoid the bactericidal activity of phagocytic cells and aids in evasion of the host immune response.

## Conclusion

The function of ALO's ability to lyse human phagocytes in *B. anthracis *pathogenesis is unclear, but this CDC may contribute to the bacteria's ability to establish, spread, or cause disease along with the other more characterized toxins. This study further supports ALO as an important virulence factor of *B. anthracis*. Not only can it act at sub-lethal concentrations with LT to induce apoptosis, but this study demonstrates that alone, at higher concentrations, ALO causes cytolysis of human phagocytes and epithelial cells. The study of other factors produced by *B. anthracis*, along with the major anthrax toxins, will lead to a better understanding of this bacterium's pathogenesis as well as provide more information for the development of anti-toxin vaccines for treating and preventing anthrax [29].

## Methods

### Growth of bacterial strains

*B. anthracis *Sterne strain 7702, a capsule-negative toxigenic (containing plasmid pXO1 but not pXO2) strain[5], was grown in Brain-Heart Infusion broth (BHI; Difco, Detroit, M.I.), without added bicarbonate, with shaking (200 rpm) at 37°C in an air shaker incubator (New Brunswick Scientific, Edison N.J.) or on BHI agar in a humidified 5% CO_2 _incubator [37]. The bacterial strains UT231, an ALO knockout, and UT231 (pUTE544), an ALO-over-expressing, *alo *complemented strain, used in this study have been previously described [8]. Growth of UT231 and UT231 (pUTE544) was supplemented with 50 μg/ml kanamycin and 10 μg/ml tetracycline, respectively. Bacteria were grown to late exponential phase (0.66 absorbance at 600 nm) and washed in PBS before addition to cell culture. Cell free supernatants were obtained after centrifugation of cultures at 10,000 rpm for 6 min and subsequent filtration through 0.22 μm MillexGP filter units (Millipore, Molsheim, France).

### Cell culture

The human monocytic cell line THP-1 (ATCC TIB-202) was maintained in RPMI with heat-inactivated 10% fetal bovine serum, FBS, (Gibco BRL), and 50 μM beta-mercaptoethanol at 37°C with 5% CO_2 _according to ATCC recommendation. Cells were used between passages 10–20. The human cervical epithelial-like cell line ME-180 (ATCC HTB-33) was maintained in McCoy's medium with heat-inactivated10% FBS according to ATCC recommendations. A549 (ATCC CCL-185), human lung epithelial cells, and Detroit 562 (D562, ATCC CCL-138), human pharyngeal epithelial cells, were maintained according to ATCC recommendation. All FBS was heat-inactivated for 30 min at 56°C.

### Primary cell isolation

PMNs were isolated from 50 ml of human blood through Ficoll-Hypaque gradient as previously described [38]. Cells were resuspended to 2 × 10^6^/ml in PBS containing 1 mg/ml gelatin (PBSG) plus 1 mM Ca^2+ ^and 1 mM Mg^2+^. Viability and purity were ≥ 95% as determined by trypan blue staining and basic cell morphology (tri-lobed nuclei), respectively.

Monocytes were isolated from total mononuclear cells from 100 ml of human buffy coat (Biological Specialty Corporation, Colmar, PA) using Ficoll-Paque Plus (Amersham Biosciences, Uppsala, Sweden) followed by enrichment of monocytes using RosetteSep Human Monocyte Enrichment Cocktail (Stem-Cell Technologies, Vancouver, BC) according to manufacturer directions. To obtain human monocyte derived macrophages (hMDMs), monocytes were resuspended in RPMI complete medium supplemented with 100 μg/ml penicillin/streptomycin (BioWhittaker, Walkersville, M.D.) and 10 ng/ml recombinant human M-CSF (Peprotech, Rock Hill, N.J.). Monocytes were seeded in 24 well cell culture treated plates (Cat no. 3524, Corning, N.Y.) containing Thermanox coverslips (Nalge Nunc, Rochester, N.Y.) at 8 × 10^4 ^cells/well and incubated at 37°C with 5% CO_2 _overnight. Wells were washed and replaced with fresh RPMI complete medium and grown for 5 days until assayed [39]. hMDMs were washed once with fresh medium before assaying viability. Viability was determined by trypan blue staining to be ≥ 95%. Cells were more than 95% macrophages as assessed by adherence to coverslips and non-specific esterase staining (α-naphthyl acetate esterase kit, Sigma) following the manufacturer's instructions. The differentiated phenotype was confirmed by flow cytometry analysis of CD71 and CD1a (BD Pharmingen, San Diego, CA) at the cell surface [40].

Enrichment of total lymphocytes was likewise conducted using RosetteSep Total Lymphocyte Enrichment Cocktail (Stem-cell Technologies, Vancouver, BC). Purity and viability were ≥ 95% as determined by trypan blue staining and expression of CD3 (BD Pharmingen, San Diego, CA) by flow cytometry analysis, respectively.

### Expression and purification of rALO

Recombinant ALO was expressed as described [8]. For use in assays rALO was diluted in phosphate-buffered saline (PBS) containing 0.1 mg/ml BSA. 1 mg/ml of cholesterol (Sigma-Aldrich) stock solution in 100% ethanol was used in cholesterol inhibition experiments at a final concentration of 1 μg/ml. Final ethanol concentration is 0.1%. In cholesterol inhibition experiments an ethanol control was used, without cholesterol, and did not affect cell lysis.

### Hemolysis assay

The assay was performed in 96-well plates. One micro liter of freshly prepared 0.6 M cysteine in PBS was added to 100 μl of 2-fold (or 10-fold) dilutions of freshly prepared bacterial supernatants and allowed to incubate at room temperature for 10 min in a 96-well round-bottom plate. After incubation, 35 μl of PBS and 15 μl of a 10% (v/v) suspension of human red blood cells washed four times with PBS were added to each well. The plate was allowed to incubate at 37°C for 1 hwith gentle and occasional rocking and then centrifuged at 700 rpm for 1 min. The results were read by placing the plate on a horizontal light box and then viewing (and photographing) the plate from above.

### Measurement of cell viability

PMNs and rALO (diluted accordingly in 0.1% BSA/PBS) were combined 1:1 in a 1.5 ml eppendorf tube and incubated with gentle tumbling, 15 rpm, at 37°C for 10 min. 10 μl of sample was then added to 10 μl of 0.25% trypan blue (ICN Biomedicals, Aurora, OH) in 0.85% NaCI. Following 5 min incubation at room temperature the percentage of viable cells was calculated by blind counting of at least 100 cells under 200× to 400× magnification. Viable cells remain colorless whereas dead cells are blue.

THP-1s were cultured in RPMI+10% FBS, washed, and resuspended in PBS to 2 × 10^6^/ml. THP-1s and rALO (diluted accordingly in 0.1% BSA/PBS) were combined 1:1 in 1.5 ml eppendorf tubes and incubated with gentle tumbling at 37°C for 10 min. Lymphocyte viability was determined as described for the THP-1 s. Monocyte-derived macrophages were washed once with fresh medium before assaying viability as described. Reduction of a tetrazolium dye was measured using the In vitro Toxicology based lactate dehydrogenase kit (Sigma-Aldrich Tox-7) according to manufacturer instructions. The percentage of cytotoxicity was calculated as [(sample OD_490 _- OD_0%_)/(OD_100% _- OD_0%_)] × 100, where OD_0% _represents the OD_490 _of cells alone and OD_100% _represents the OD_490 _of lysed cells. Percent viability was calculated by subtracting percent cytotoxicity from 100. The validity of the LDH assay was confirmed by comparison with results obtained from the trypan blue exclusion assay.

### Statistical analyses

A p-value that was < 0.05 was considered statistically significant. The affect on increasing dose of ALO on cell cytotoxicity was evaluated for statistical significance using Spearman's correlations. Individual samples at each concentration served as the unit of analysis. The difference in the ability of ALO to lyse lymphocytes in the presence or absence of serum was analyzed for significance by using student's un-paired t-test (two-tailed p-value). Differences in viability due to strain (7702, UT231, UT231 (pUTE544) and dose were analyzed for significance using 2 factor analysis of variance (ANOVA). Differences in viability due to strain (7702, UT231, UT231 (pUTE544) were analyzed for significance using 1 factor analysis of variance (ANOVA). We chose to analyze MOI of 100 and MOI of 10, as there is the greatest ambiguity of differences in viability between strains at these MOIs. 1 and 2 way ANOVAs were followed up with Tukey tests to show differences between each strain. rALO ED_50_s calculated based upon dose-response curve extrapolation. The SPSS v. 14 software (SPSS, Chicago, IL) was used for these analyses.

## Authors' contributions

EMM carried out the experiments and drafted the manuscript. RFR participated in the design of the study and helped to draft the manuscript. All authors read and approved the final manuscript.

## Figures and Tables

**Table 1:** Recombinant ALO ED_50 _in the absence of serum. Results show amount of rALO necessary for 50% cytolysis of primary human PMNs, lymphocytes, monocyte-derived macrophages, THP-1 monocytes, ME-180, D562, and A549 epithelial cells after 30 min at 37°C. * PMN cytolysis was measured at 10 min. Data derived from Figures 1–5 by cytotoxicity curve extrapolation.

	rALO ED_50_	
	ng/ml	nM
PMNs*	4	0.070
THP-1 monocytes	20	0.35
Lymphocytes	25	0.44
MDMs	100	1.8
ME-180	1000	18
D562	1000	18
A549	50	0.88

**Table 2:** ALO-mediated lysis of human monocytes by *B. anthracis *is inhibited by 10% FBS. Percent viable THP-1s in the presence of *B. anthracis *Sterne strain 7702 (ALO+), UT231 (ALO-) and the complemented over-producer, UT231 (pUTE544) (ALO++) at an MOI of 1000 in the presence of 10 % FBS. At various time points, aliquots were mixed with 0.25 % trypan blue to measure viability. Native ALO expressed by 7702, at all time points, and UT231 (pUTE544), at 5 min, is inhibited by cholesterol present in heat-inactivated 10% FBS. Results are average ± standard deviations of two experiments.

**% Viable THP-1 monocytes in presence of 10% FBS**				
	**Sterne 7702**	**UT231**	**UT231(pUTE544)**	**Control (no bacteria)**
**5 min**	100	100	100	100
**30 min**	100	100	0	99 ± 0
**60 min**	95 ± 1	98 ± 2	0	99 ± 0
**120 min**	92 ± 3.2	96 ± 1	0	97 ± 1
